# Comparative chloroplast genome analysis of seven extant *Citrullus* species insight into genetic variation, phylogenetic relationships, and selective pressure

**DOI:** 10.1038/s41598-023-34046-6

**Published:** 2023-04-25

**Authors:** Cong Zhou, Putao Wang, Qun Zeng, Rongbin Zeng, Wei Hu, Lei Sun, Shi Liu, Feishi Luan, Qianglong Zhu

**Affiliations:** 1 Jiangxi Key Laboratory of Crop Physiology, Ecology and Genetic Breeding, Jiangxi Agricultural University, NO. 1101 Zhimin Street, Qingshanhu District, Nanchang, 330045 People’s Republic of China; 2 College of Horticulture and Landscape Architecture, Northeast Agricultural University, Harbin, 150030 People’s Republic of China; 3 Department of Agronomy and Horticulture, Liaoning Agricultural Technical College, Yingkou, 115009 People’s Republic of China

**Keywords:** Genetics, Molecular biology, Plant sciences

## Abstract

*Citrullus ecirrhosus*, *Citrullus rehmii*, and *Citrullus naudinianus* are three important related wild species of watermelon in the genus *Citrullus*, and their morphological differences are clear, however, their chloroplast genome differences remain unknown. This study is the first to assemble, analyze, and publish the complete chloroplast genomes of *C. ecirrhosus*, *C. rehmii*, and *C. naudinianus*. A comparative analysis was then conducted among the complete chloroplast genomes of seven extant *Citrullus* species, and the results demonstrated that the average genome sizes of *Citrullus* is 157,005 bp, a total of 130–133 annotated genes were identified, including 8 rRNA, 37 tRNA and 85–88 protein-encoding genes. Their gene content, order, and genome structure were similar. However, noncoding regions were more divergent than coding regions, and *rps16-trnQ* was a hypervariable fragment. Thirty-four polymorphic SSRs, 1,271 SNPs and 234 INDELs were identified. Phylogenetic trees revealed a clear phylogenetic relationship of *Citrullus* species, and the developed molecular markers (SNPs and *rps16-trnQ*) could be used for taxonomy in *Citrullus*. Three genes (*atpB*, *clpP1*, and *rpoC2*) were identified to undergo selection and would promote the environmental adaptation of *Citrullus*.

## Introduction

The genus *Citrullus* contains approximately seven extant species, *Citrullus lanatus*, *Citrullus mucosospermus*, *Citrullus amarus*, *Citrullus ecirrhosus*, *Citrullus rehmii*, *Citrullus colocynthis*, and *Citrullus naudinianus*^1,2^. *C. lanatus* subsp. *vulgaris* (Watermelon) is a worldwide cultivated horticultural crop bearing large fruits with crisp sweet, juicy, and red flesh and a green rind with black stripes. Its fruit often contains over 90% water and many nutritional compounds, including sugars, carotenoids and amino acids for cardiovascular prevention^3^, which makes it one of the most popular fruit crops around the word. Recent research suggested that the center of diversity and origin of modern watermelon, which is now cultivate and consume all over the world, is Kordofan in Sudan^2^. In addition to s *Citrullus lanatus* subsp*. vulgaris*, the genus *Citrullus* comprises six other cultivated or wild species, *C. naudinianus* is the most primitive wild species, and it is diecious and widely distributed in sub-Saharan Africa^4^. *C. colocynthis* is used for folk medicine with therapeutic effects for various diseases, such as antidiabetic and anticancer effects^5–7^, and mainly originates from northern Africa. *C. rehmii* and *C. ecirrhosus* commonly grow in desert climates and environments, and they are commonly found in southern Africa^1^. *C. amarus* is a citron watermelon that can be directly used as a water source and be processed into jam and feedstuff, and it originated from southern Africa and spread to the Mediterranean region^8^. *C. mucosospermus* is an egusi watermelon with large edible seed and is native to western Africa^2^. Furthermore, these wild species have been broadly applied to explore phylogenetic relationships and genetically improve cultivated watermelon (*C. lanatus* subsp*. vulgaris*)^1,2,8,9^.

The chloroplast is an essential semi-autonomous organelle that mainly conducts photosynthesis and is important for the growth and development of plants and green algae. Chloroplasts are also responsible for the synthesis of starch, fatty acids, amino acids and other metabolites, and play an important role in the stress response^10–12^. Chloroplasts possess a circular double-stranded DNA molecule. The complete chloroplast genome (CPG) usually demonstrates a relatively conserved quadripartite structure including a large single copy (LSC) and a small single copy (SSC) region, and two inverted repeats (IRa and IRb). Compared with nuclear genomes, the chloroplast genome has a unique pattern of maternal inheritance, a large number of genes and a lower mutation rate in adaptive evolution. Therefore, CPGs have been proposed as molecular markers to identify and classify plant species by the Consortium for the Barcode of Life (CBOL)^13^. The first chloroplast genome sequencing of tobacco was completed in 1986. By September 2022, there were 8,210 complete plant chloroplast or plastid genomes deposited in genome database of the National Center for Biotechnology Information (NCBI), comprising ~ 315 genes (encoding proteins, transfer RNA and ribosomal RNA) ranging from 15 to 707 kb in size. The chloroplast reference genomes of *C. lanatus* subsp*. vulgaris*, *C. amarus* and *C. colocynthis* have been published in our past researches^14–16^. And the chloroplast reference genome of *C. mucosospermus* was also published in 2017^17^. To explore the phylogenetic relationship within the genus *Citrullus* and especially the genetic variation of chloroplast genomes during the selection and domestication of cultivated watermelon, there is an urgent need for more chloroplast genomes of other *Citrullus* species to be published.

Here, we aimed to assemble and analyze the chloroplast genomes of three wild watermelon species, *C. rehmii*, *C. ecirrhosus* and *C. naudinianus*, based on their whole genome sequence data stored in the Sequence Read Archive (SRA) database of NCBI. We next compared their chloroplast genomes with the published CPGs of four other *Citrullus* species. Our study aims were: (1) to publish the CPGs of *C. ecirrhosus*, *C. rehmii*, and *C. naudinianus*, (2) to reveal the structural features of seven CPGs and evaluate the genetic variation in the chloroplast genome of *Citrullus*, and (3) to identify the molecular markers and the genes under positive selection, explore the phylogenetic relationships of seven *Citrullus* species, with the hope of providing insights into the speciation, selection and domestication of cultivated watermelon.

## Results

### Chloroplast genome generation, annotation, and comparative feature analysis

The sequence data (0.8 Gb) of *C. ecirrhosus*, *C. rehmii*, and *C. naudinianus* were extracted randomly, and approximately 13.27% (*C. ecirrhosus*), 21.37% (*C. rehmii*) and 12.75% (*C. naudinianus*) of these clean paired reads mapped to the CPG of C. lanatus subsp. vulgaris. The CPGs of these three Citrullus species were obtained after de novo assembly and strict validation. Then, they were annotated and archived in GenBank with accession number ON597627, MZ577999, and MZ577998.

**Figure 1:**
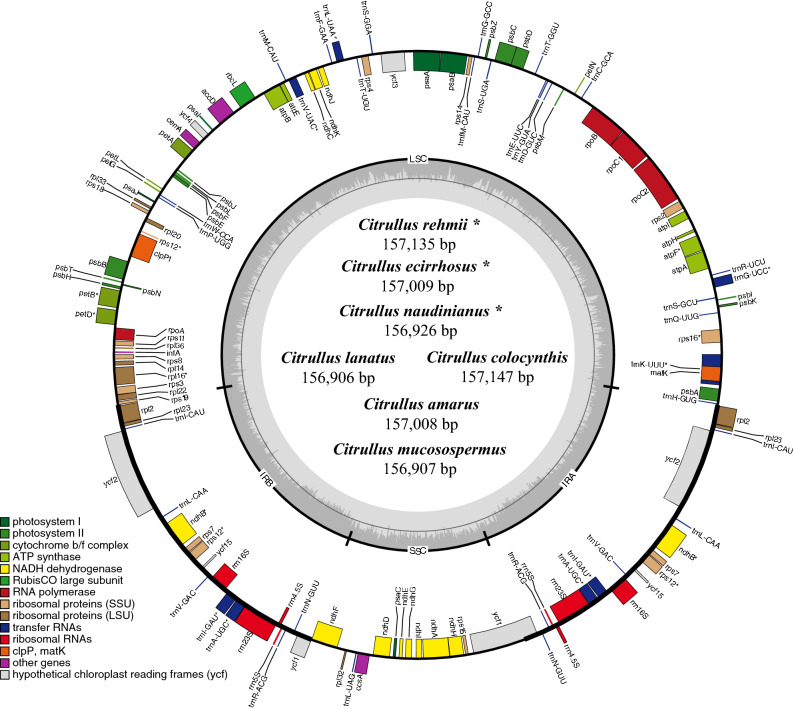
Complete chloroplast genome circle map of the seven studied *Citrullus* species. Genes outside the circle are transcribed on the positive chain, whereas those genes inside the circle are transcribed on the negative chain. Boxes with different colors represent genes with a different functional group. The inner ring shows the positions of LSC, IRa, SSC, and IRb, with the GC clamp of the chloroplast genome shown in grey. The chloroplast genome map was draw using OGDRAW v1.3.1 (https://chlorobox.mpimp-golm.mpg.de/OGDraw.html).

To reveal the structural features of seven CPGs in the genus *Citrullus*, comparative analysis was conducted (Table 1), The average length of the CPGs is 157,005 bp, ranging from 156,906 bp (*C. lanatus* subsp*. vulgaris*) to 157,147 bp (*C. colocynthis*), and they demonstrated a typical quadripartite structure, including the LSC (86,728–87,007 bp) and SSC (17,897–17,999 bp) regions, and a pair of IRs (26,072–26,149 bp), but the variation coefficient of the SSC regions (2.57E−3) was greater than that of the LSC regions (9.56E−4) and IR regions (1.42E−3). The average GC content (%) in the seven CPGs was approximately 37.16. Further analysis of GC content found that the IR region had the highest GC content compared to any other region in each CPG (Table 1). When the gene content of chloroplast genomes was compared, we found some mistakes in the original annotation of *C. lanatus* subsp*. vulgaris* and *C. mucosospermus*, then we updated the chloroplast genome annotation of *C. lanatus* subsp*. vulgaris* at NCBI. After correcting the chloroplast genome annotation of *C. lanatus* subsp. vulgaris and *C. mucosospermus*, we found that the seven CPGs encoded a set of 130–133 functional genes, including 85–88 protein-encoding genes, 37 transfer RNAs (tRNA), and 8 ribosomal RNAs (rRNA). Furthermore, 79 protein-encoding genes were identified among seven *Citrullus* species. Twenty-three genes were found to harbor introns, four of which (two *rps12* gene, one *clpP* gene, and one *ycf3* gene) had two introns. A consensus radius map of the seven CPGs was plotted to explore the variation in the IR/SC boundary (Supplementary Fig. S1), suggested that there was no the significant variation in the IR/SC boundary. Moreover, fine collinearity was observed among the seven CPGs of Citrullus species (Supplementary Fig. S2). Based on the conclusion that the seven CPGs have similar total gene numbers, names, and orders, their annotated CPGs of the seven species were drawn in a circle map (Fig. 1).

**Table 1:** Summary statistics for the assembly of seven CPGs in the genus *Citrullus*.

Genome features	C. ecirrhosus*	C. naudinianus*	C. rehmii*	*C. lanatus* subsp.* vulgaris*	*C.colocynthis*	*C. amarus*	*C.mucosospermus*	Average ± SD	variation coefficient
Size (bp)	157,009	156,926	157,071	156,906	157,147	157,008	156,907	157,005 ± 84	5.36E−4
LSC length (bp)	86,823	86,728	86,887	86,845	86,852	86,813	86,846	86,845 ± 46	5.32E−4
SSC length (bp)	17,890	17,908	17,886	17,897	17,999	17,897	17,897	17,925 ± 36	2.05E–3
IR length (bp)	26,148	26,145	26,149	26,082	26,148	26,149	26,082	26,118 ± 29	1.13E−3
Genes number	133	133	133	131	131	130	131	132	0
Protein-coding genes	88	88	88	86	86	85	86	87	0
tRNA	37	37	37	37	37	37	37	37	0
rRNA	8	8	8	8	8	8	8	8	0
Overall GC content (%)	37.18	37.12	37.16	37.18	37.14	37.17	37.18	37.16 ± 0.02	–
GC content in LSC (%)	34.96	34.89	34.94	34.94	34.90	34.95	34.95	34.93 ± 0.03	–
GC content in SSC (%)	31.53	31.43	31.52	31.54	31.47	31.52	31.54	31.51 ± 0.04	–
GC content in IR (%)	42.80	42.76	42.80	42.83	42.82	42.81	42.83	42.81 ± 0.02	–
Accession no. in GenBank	MZ577999	MZ577998	ON597627	NC_032008/KY014105	NC_035727/MF357889	NC_035974/MF536694	NC_033899/KY430686	–	–

*LSC* Large single copy, *SSC* small single copy, *IR* inverted repeat.

*Indicated the chloroplast genome of species was assembled in the study.

### Chloroplast genome sequence divergence and nucleotide diversity analysis

**Figure 2:**
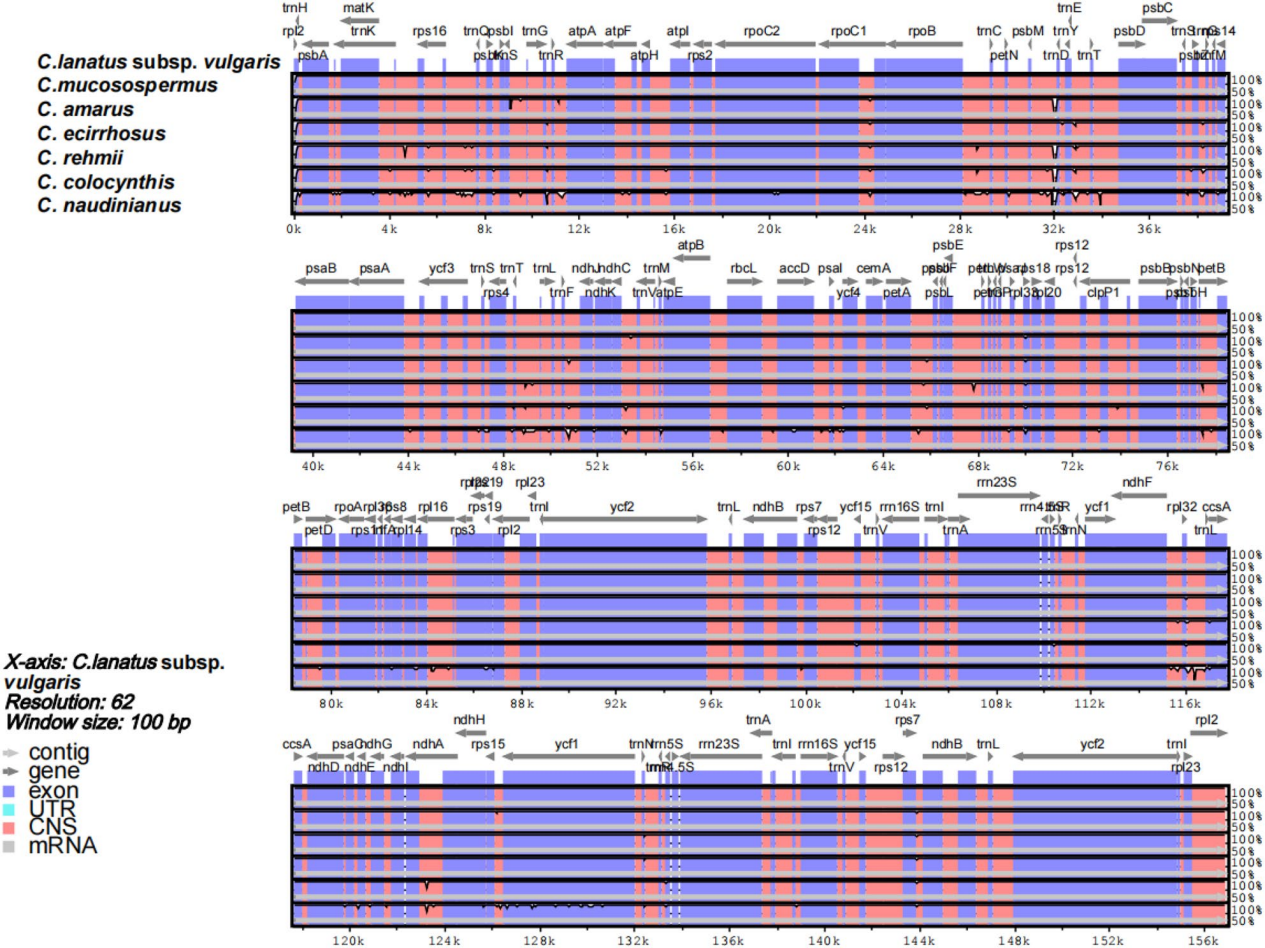
Structure comparison of seven CPGs using mVISTA program.

The CPGs of *C. rehmii*, *C. ecirrhosus*, and *C. naudinianus* were aligned with those of four other four *Citrullus* species using mVISTA and MEGA software with the CPG of *C. lanatus* subsp*. vulgaris* as a reference. The results showed that intergenic or noncoding regions were more divergent than gene encoding regions, and the largest sequence divergence was found between *C. lanatus* subsp*. vulgaris* and *C. naudinianus*, the smallest divergence was found between *C. lanatus* subsp*. vulgaris* and *C. mucosospermus* (Fig. 2). Their sequence divergence was also validated by the pairwise distance. The pairwise distances among the seven *Citrullus* species ranged from 0.0004 to 0.00641 (Supplementary Table S1). The largest sequence divergence of 0.00641 was found between *C. naudinianus* and *C. lanatus* subsp*. vulgaris* or *C. mucosospermus*, whereas the smallest sequence divergence of 0.0004 was found between *C. lanatus* subsp*. vulgaris* and *C. mucosospermus*.

**Figure 3:**
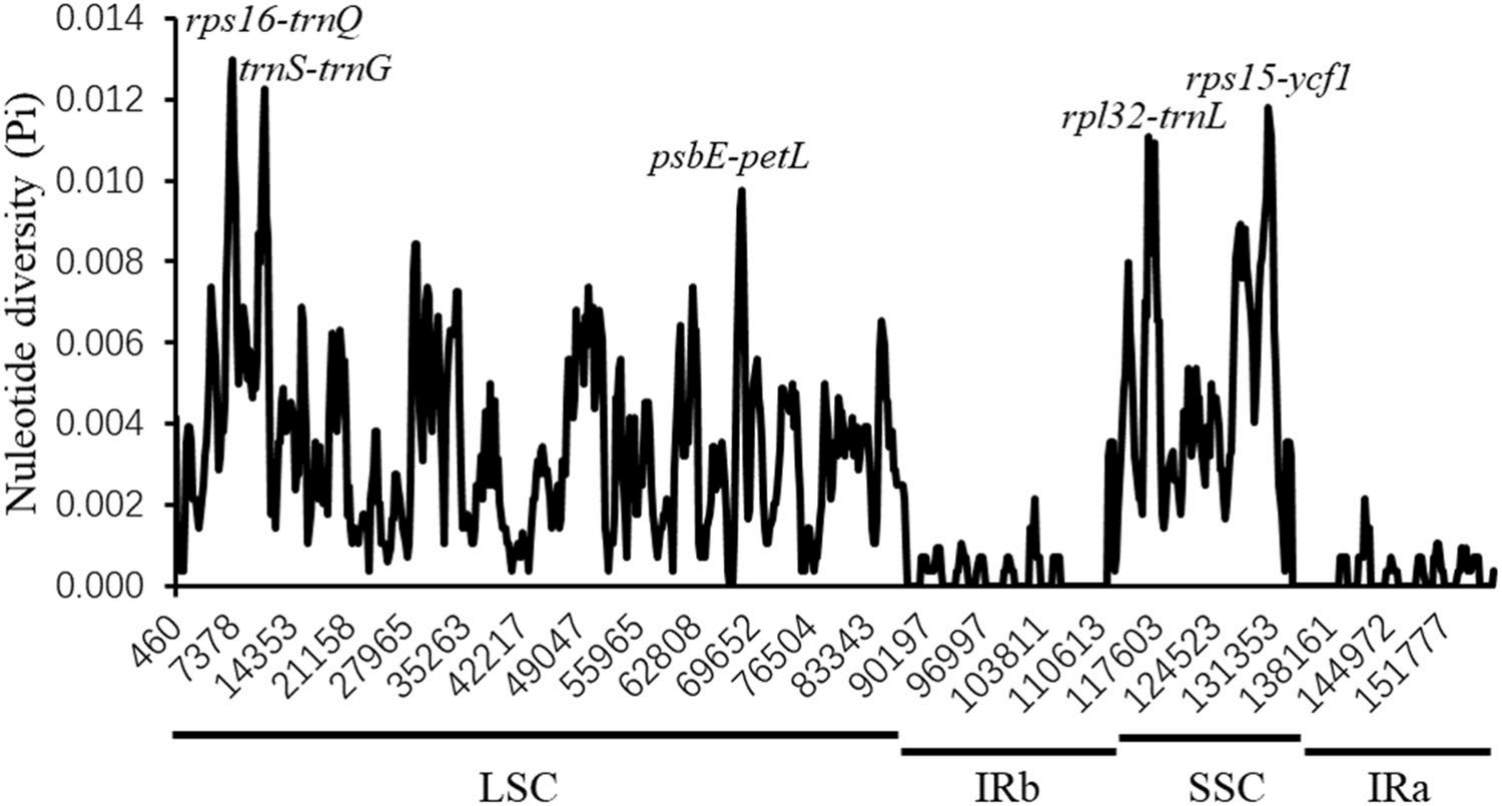
Position of the midpoint in the window Sliding window plots of nucleotide diversity (pi) across the complete chloroplast genome of the seven *Citrullus* species. Window length: 800 bp; Step size: 200 bp.

To explore the nucleotide diversity (Pi value) at the different regions of the CPGs, the nucleotide diversity of the seven CPGs and the consensus protein coding regions in the *Citrullus* genus were calculated by DnaSP, and the data plot showed that the LSC and SSC regions were more divergent than the two IR regions (Fig. 3). The intergenic regions (*rps16-trnQ* (0.013), *trnS-trnG* (0.012), *rps15-ycf1* (0.012), *rpl32-trnL* (0.011), and *psbE-petL* (0.010)) were the top five highest variables among the noncoding regions (Table 2), but the highest Pi value of all the protein gene coding regions was 0.00754 (*rpl32*), which was less than that of most noncoding regions (Supplementary Table S2). After phylogenetic relationship analysis based on the five hypervariable fragments (Supplementary Fig. S3), we found that all bootstrap values on the phylogenetic tree of *rps16-trnQ* were greater than 75%, and the topological structure was the same as that in previous studies, showing that the successful identification ratio of *rps16-trnQ* is 100%, while the other four hypervariable fragments had low success identification ratios, suggesting that only *rps16-trnQ* could be used as a high-efficiency molecular marker or DNA barcode for *Citrullus* species identification.

**Table 2:** Comparative analysis of five hypervariable fragments in *Citrullus*.

DNA barcode	Location ^a^	Aligned length	Pi	VS	PIn	K-2P distance	DSR (%)
*rps16-trnQ*	6538–7748	1222	0.013	40	9	0.011	100
*trnS-trnG*	10,397–11,437	1091	0.012	36	6	0.012	25
*psbE-petL*	67,227–68,042	816	0.010	24	4	0.010	50
*rpl32-trnL*	115,354–116,867	1556	0.011	40	11	0.010	75
*rps15-ycf1*	129,498–130,696	1199	0.012	40	6	0.011	75

^a^The location of five hypervariable fragments is confirmed based on the chloroplast genome of *C. lanatus* subsp*. vulgaris.*

*VS* Variable sites, *Pin* Parsimony informative sites, *DSR* Discrimination success ratio.

#### Repeat sequence analyses

**Figure 4:**
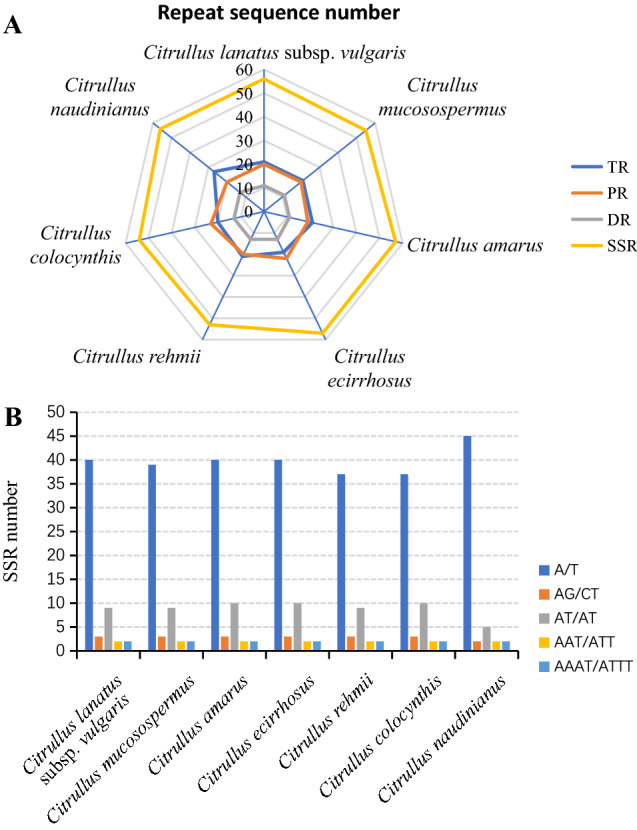
Different repeat sequence number showed in Radar-plot (**A**), Number of SSRs and types detected in the seven CPGs of *Citrullus* (**B**).

Repeat sequences are commonly used as an important molecular source for understanding species phylogenetic relationships, yet they can also act a significant role in genome rearrangement^18,19^. We detected TR, PR, DR, and SSRs in all seven *Citrullus* species chloroplast genomes. The CPGs of the seven *Citrullus* species contained 107–116 repeat sequences in total (Fig. 4A, Supplementary Table S3), including 20–27 TRs, 19–23 PRs, 11–13 DRs, and 53–57 SSRs; notably, the TR number of *C. naudinianus* (27) was the highest, making the total number of repeat sequences in *C. naudinianus* (116) significantly greater than those in the other species, while the number of repeat sequences was not significantly different among the other *Citrullus* species. Many repeat sequences were mainly distributed within the noncoding regions (intergenic and intron regions) of the seven CPGs (Supplementary Table S4).

**Figure 5:**
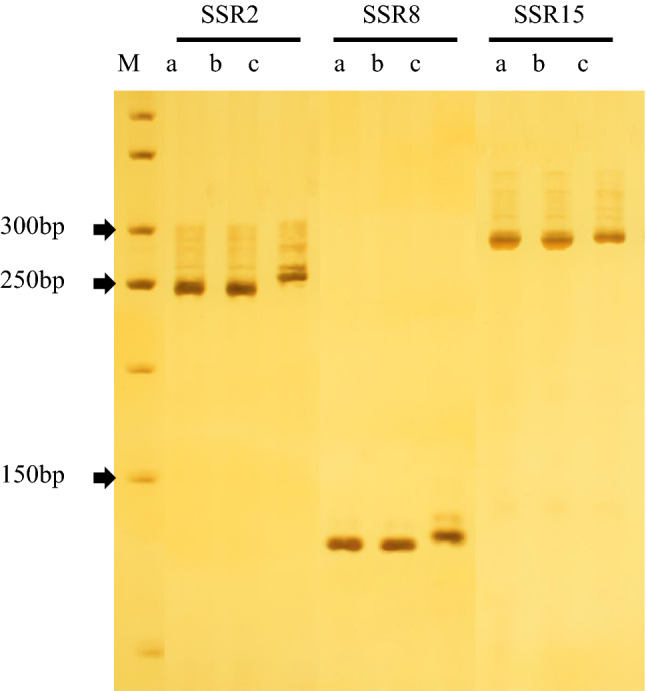
Three selected SSR makers were polymorphic among the *C. lanatus* subsp*. vulgaris* (W1-1), *C. mucosospermus* (PI186490) and *C. amarus* (PI269341), M: DL500 DNA marker, a: W1-1, b: PI186490, c: PI269341.

The average SSR number was 56 (Supplementary Table S5). Mono-, di-, tri-, tetra-, penta-, and hexa-nucleotide SSRs in each species were analyzed, but only mono-, di-, tri-, and tetra-nucleotide SSRs were found (Fig. 4B), and the average number and proportions of mono-, di-, tri-, and tetra-nucleotide SSRs were 40(71.6%), 12(21.1%), 2(3.6%), and 2(3.6%), respectively (Supplementary Table S5). Notably, no penta- or hexa-nucleotide SSRs were found in the seven CPGs of *Citrullus*, and the number of mononucleotide SSRs in *C. naudinianus* (45) was the highest among the seven species. Nevertheless, the SSRs in these chloroplast genomes were remarkably rich in A/T content, with lower G/C content. The A/T mononucleotide repeats constituted a large portion of the SSRs in all species. In addition, we found that the seven species shared 47 SSRs, and 34 of those SSRs were polymorphic (Table S6). Three selected SSR markers were polymorphic among *C. lanatus* subsp*. vulgaris*, *C. mucosospermus* and *C. amarus* (Fig. 5, Supplementary Fig. S4), suggesting that these SSR markers could be applied for species classification in *Citrullus*.

#### Chloroplast genome variation map

**Figure 6:**
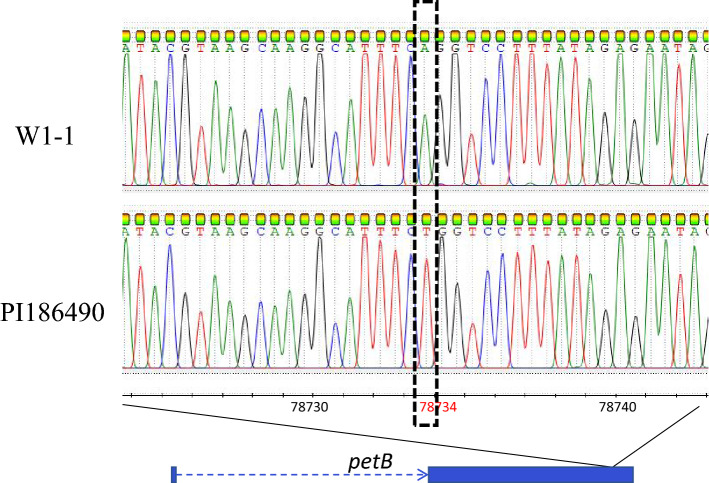
Targeted pyrosequencing assays of petB in W1-1 (*C. lanatus* subsp*. vulgaris*) and PI186490 (*C. mucosospermus*).

To identify the variation in the chloroplast genome between cultivated watermelon and the other six *Citrullus* species, 1271 single nucleotide polymorphisms (SNPs) were identified using VarScan, of which 769 (61.5%) SNPs were in noncoding regions, such as introns, and upstream and downstream regions of genes, and approximately 39.5% of SNPs were in coding regions, which led to synonymous variants and missense variants (Table 3). Notably, except for synonymous variants, 242 (19.0%) SNPs in coding regions resulted in variants of 43 protein coding genes (Supplementary Table S7). Of these genes, the *accD*, *clpP*, *ndhF*, *rpoC2*, *ycf1*, and *ycf2* harbor more than 10 missense SNPs. Notably, only four SNPs were identified between cultivated watermelon and its nearest wild species (*C. mucosospermus*), and only one SNP (A/T at loci 78,734) was identified that altered the CDS of *petB*, which was validated by targeted pyrosequencing assays (Fig. 6), other three SNPs were in noncoding regions.

**Table 3:** The number of SNP effects with different type and region.

Type and region	Counts	Percent (%)
Upstream gene variant	761	59.9
Synonymous variant	260	20.5
Missense variant	242	19.0
Splice region variant and stop retained variant	1	0.1
Splice region variant and intron variant	3	0.2
Downstream gene variant	1	0.1
Stop gained	2	0.2
Stop lost and splice region variant	1	0.1

We also identified 234 small insertions or deletions (INDELs) among the chloroplast genomes of seven species. Most INDELs were in the intergenic regions and introns, and only 9 (3.8%) INDELs were located in six protein-coding regions of the *matk*, *rpl33*, *infA*, *rps19*, and *ycf1* genes (Supplementary Table S8). Notably, *matk*, *infA*, and *rps19* contain both variations, and a total of 45 protein-coding regions were altered by SNP or INDEL.

#### Phylogenetic relationships analysis

**Figure 7:**
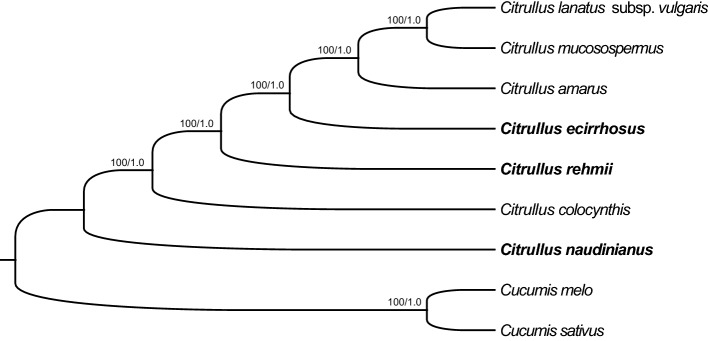
ML tree showing phylogenetic relationships among seven *Citrullus* species conducted using complete chloroplast sequences. *Cucumis sativus and Cucumis melo* acted as the out-group. The corresponding ML-BS/BI-PP values are given at each node. *C. ecirrhosus*, *C. rehmii*, and *C. naudinianus* were marked in bold characters.

The phylogenetic relationships of seven *Citrullus* species were analyzed with the ML and BI methods based on the CPG, CDS, SNP and DNA barcode, and the resulting topologies of the seven cucurbit plants were largely congruent, with highly supported values (Fig. 7 and Supplementary Fig. S5). Figure 7 shows the phylogenetic tree of CPG generated by the ML analysis, including two types of supporting values: the ML bootstrap (ML-BS), and BI posterior probability (BI-PP). All ML-BS and BI-PP values on the clade were 100 and 1.0, respectively. The phylogenetic tree revealed that the seven *Citrullus* species were grouped into a clade, and the *C. naudinianus* was at the base of the clade, followed by *C. colocynthis, C. rehmii, C. ecirrhosus, C. amarus, C. mucososperms*, and *C. lanatus* subsp*. vulgaris*.

#### Selective pressure events

The ratio (ω) was calculated to evaluate the selective pressure on the 79-consensus protein-encoding genes of the seven CPGs of *Citrullus*. Three genes (*atpB, clpP,* and *rpoC2*) were detected to be subject positive to selective pressure by EasyCodeML (Table 4). The ω in the M2a model (ω2) changed from 177.5821 to 429.3408, and of the three genes subject to positive selective pressure, *clpP* had the greatest ω2 value, which indicated that *clpP* could have undergone intense positive selection. Meanwhile, the amino acid sites in the three genes under positive selective pressure were determined, revealing that these genes harbor one intensively positive selective site (Table 4). Further analysis using the Ka/Ks calculator in Tbtools showed that the *clpP1* gene was positively selected (ω > 1) between *C. lanatus* subsp*. vulgaris* and *C. naudinianus* (Supplementary Table S9).

**Table 4:** The summary of positive selective pressure analysis on genes in M2a, M7 vs. M8 model.

Gene name	Model	np	LnL	ω2(M2a)	LRTs(2△LnL)	LRT* p*-value	Positive sites
*atpB*	M8	16	− 2114.47	197.5187	38.81554	4.00E−09	52 Q*
	M7	14	− 2133.88				
*clpP*	M8	16	− 890.821	429.3408	34.30236	3.60E−09	108 S**
	M7	14	− 907.973				
*rpoC2*	M8	16	− 5901.01	177.5821	52.56322	0	937 G*
	M7	14	− 5927.29				

*Means *p* < 0.05, **means *p* < 0.01.

## Discussion

In our study, the CPGs of three species of the genus *Citrullus* were de novo assembled and annotated followed by a comparative analysis with four other *Citrullus* species, all methods were strictly conducted according to the above relevant experimental methods. The chloroplast genome size of the seven *Citrullus* species was 157,005 ± 102 bp, suggesting that their genome size changed little over the course of evolution. The length change of the SC and IR regions are the dominant factor contributing to the differentiation of CPG size, the SSC regions of *Citrullus* have the highest variable coefficient among the SC and IR regions, and the correlation coefficient with CPG size was 0.87 (R^2^) in our study, indicating that the variations of chloroplast genome size result from the expansion and contraction of SSC regions in *Citrullus*. The CPGs of *Citrullus* were composed of eight rRNA, 37 tRNA and 85–88 protein-encoding genes, and similar gene contents and types have been reported in other plants in Cucurbitaceae^20–22^. The noncoding regions showed higher sequence divergence than the protein-encoding regions, suggesting that most sequence variation in the noncoding regions could be further exploited and utilized.

Repeated sequences could play a significant role in producing variation and rearranging genomes in CPG^23^. The TR, PR, DR, and SSRs were analyzed in the CPGs of seven *Citrullus* species in our research, a lot of repeat sequences were located in the intergenic regions of the *Citrullus* CPGs, the resemble results to our study have been found in the CPGs of Prunus and Quercus^10^. The TR and mono SSR numbers in the chloroplast genome of *C. naudinianus* were greater than those in the other *Citrullus* species, which could contribute to the genetic divergence between *C. naudinianus* and other *Citrullus* species. Polymorphic SSRs in the chloroplast genome have been applied to study on the population genetics and evolutionary of *Citrullus*^24^, in our study, Thirty-four SSRs were detected to be polymorphic among *Citrullus* species, suggesting our identified polymorphic SSRs could be developed to study on the genetics and evolutionary in *Citrullus*.

The SNPs and INDELs on the chloroplast genome have been used to study the diversity existed at the intra-species and inter-subspecies level^25–28^. A total of 1271 SNPs were identified in the CPGs of seven *Citrullus* species, approximately 1 SNP/123 bp, which is less than that in the nuclear genome of 1 SNP/18 bp^1^, suggesting that the mutation rate of the chloroplast genome is lower than that of the nuclear genome. Forty-three protein coding genes have been changed by 19.0% SNPs in the coding region, especially, the *accD*, *clpP*, *ndhF*, *rpoC2*, *ycf1*, and *ycf2* harbor more than 10 missense SNPs. These genes play a key role in plant photosynthesis and energy metabolism, but further research is needed to better understand the differences resulting from their mutations among *Citrullus* accessions. Although distinct differences in phenotype were observed between *C. lanatus* subsp*. vulgaris* and *C. mucosospermus*, they have only four SNPs at their CPGs, further supporting that *C. mucosospermus* is the most closely related wild species^1^.

The high nucleotide diversity of noncoding regions and coding regions could be developed as molecular markers or DNA barcodes for species identification, taxonomy and phylogenetic relationship analysis^29^. Six intergenic regions (*trnS-trnG*, *rps16-trnQ*, *rps15-ycf1*, *rpl32-trnL*, and *psbE-petL*) with the highest nucleotide diversity (Pi) were identified in our study, while only *rps16-trnQ* could be used to develop specific DNA barcodes for the identification of germplasm resources in *Citrullus*. However, *rps16-trnQ* has not been used in the *Citrullus* taxonomy related study, but it has been widely used as a high-effect molecular marker and DNA barcode in other plant families^28,30^. The *matk* and *rbcL* genes are commonly used as traditional DNA barcodes for Cucurbitaceae and other land plants^31–33^, but their Pi values were 0.0021 and 0.0012, respectively, and most genes analyzed in this study have higher Pi values (Table S2), suggesting that *matk* and *rbcL* are not the best choice for using as DNA barcodes in *Citrullus*.

The CPG is an important molecular resource with better value for probing phylogenetic relationships than the whole nuclear genome in most plants^10^. Phylogenetic analysis based on the CPG has been used to reveal the phylogenetic relationships of species in Cucurbitaceae^22,23,34^. Phylogenetic trees were built in our study based on the CPG, CDS, SNPs and DNA barcode (*rps16*-*trnQ*) and showed that *C. naudinianus* was sister to the other six species, followed by *Citrullus colocynthis*, *Citrullus rehmii*, *Citrullus ecirrhosus*, *Citrullus amarus*, *Citrullus mucososperms*, and *Citrullus lanatus* subsp*. vulgaris*, which was similar to the previously reported *Citrullus* phylogeny constructed based on phenotypic traits, ancient literature, murals, traditional chloroplast DNA barcode, and molecular markers of the nuclear genome^1,2,35–39^, indicating that the phylogenetic tree based on our data has have high reliability for resolving the relationship of the seven species, and the molecular markers (SNPs and DNA barcodes) identified in our study have potential to be used to identify and classify species in *Citrullus*. In addition, the completed chloroplast genome of *C. rehmii* (PI632755) was published in the NCBI genome (NC_035975) in a previous study (Unpublished), when *C. rehmii* (PI632755) was added to the phylogenetic analysis, the results showed that PI632755 is closer to *C. colocynthis* than to *C. rehmii* (Supplementary Fig. S6), suggesting that PI632755 could be classified as *C. colocynthis*, although it is still considered to be *C. rehmii* at U.S. National Plant Germplasm System and many previous studies^1,40^.

Selective pressure analysis of genes could provide an important insight into the adaptive evolution of species. The KA/KS ratio is commonly applied to estimate the impacts of natural selection on creatures, and how the gene mutations promote the adaptive evolution of species^22,41^. In the present study, three genes (*atpB*, *clpP1*, and *rpoC2*) that were subjected to positive selection. Among them, *atpB* encodes the ATP synthase CF1 beta subunit and plays an essential role in photosynthesis. The increasing requirements for light in watermelon inflicted strong selective pressures on genes involved in photosynthesis during plant adaptive evolution, which supports our finding of positive selection for *atpB*. the similar conclusions were also reported for other species in Cucurbitaceae^22,34^. The product of the *clpP* gene is an ATP-dependent clp protease, which has been reported to participate in the transformation of chloroplast protein and shoot development^12,22^. The fact that *clpP* underwent positive selection in our study could be related to the adaptive evolution of the vining feature like other species in Cucurbitaceae^22^. Similarly, *rpoC2* is validated to encode RNA polymerase β and could regulate the pollination process and sex differentiation in the genus *Siraitia* (Cucurbitaceae)^22^ and *sorghum*^42^. *Citrullus naudinianus* is dioecious, and the other species in *Citrullus* are monoecious^1^, so the *rpoC2* mutation could be essential for the adaptive evolution of sex differentiation in *Citrullus*. Therefore, these chloroplast functional genes could not only participate in energy metabolism, but also in plant organ development and sex differentiation.

## Conclusion

The present research reported the CPGs of *Citrullus rehmii*, *Citrullus naudinianus*, and *Citrullus ecirrhosus*, which were valuable resources for understanding the genus *Citrullus*. The CPG average length of *Citrullus* is 157,005, and all CPGs are composed of 130–133 genes. The CPG of the seven *Citrullus* species studied here showed a good level of similarity in terms of gene content, gene order and genome structure. However, the contraction and expansion of the SSC regions contribute to changes in the chloroplast genome size of *Citrullus*. The greatest sequence divergence was observed between *C. naudinianus* and *C. lanatus* subsp*. vulgaris* or *C. mucosospermus*, whereas the smallest sequence divergence was between *C. lanatus* subsp*. vulgaris* and *C. mucosospermus*. Thirty-four polymorphic SSRs, 1,271 SNPs and 234 INDELs were identified, and few variations in the coding regions led to the variation of 45 protein-coding genes. The *rps16*-*trnQ* can be developed as a high-resolution DNA barcode beneficial for taxonomy in *Citrullus*. Phylogenetic relationship analysis indicated that the clear phylogenetic relationship of *Citrullus* and molecular markers developed in our study could be applied to the species identification and classification of *Citrullus*. Three protein-coding genes (*atpB*, *clpP1*, and *rpoC2*) were subjected to selection pressure, which would promote the adaptation of *Citrullus* to the growing environment. Our research results provide a basis for understanding the genetic differences in the chloroplast genome of *Citrullus* and the domestication of cultivated watermelon.

## Materials and methods

### Plant material

Healthy fresh leaves were collected from young plants of *C.* lanatus subsp. *vulgaris* (W1-1)*, C. mucosospermus* (PI186490), and *C. amarus* (PI269341) at the horticultural station of teaching and experimentation of Jiangxi Agricultural University (JXAU) in the spring of 2022. The total DNA of these plant materials were isolated from about 3 g of young leaves using a modified cetyltrimethylammonium bromide (CTAB) method. We used agarose gel electrophoresis (1%, 180 V for 45 min) and an Agilent BioAnalyzer 2100 (Agilent Technologies Inc., California, America) to evaluate the DNA quality and quantity. Approximately 300 μg of total DNA from each sample was stored at -80 ℃ until use. Our experimental research and field studies on plants, including the collection of plant material, comply with the guidelines of JXAU and relevant institutional, national, and international guidelines and legislation.

### Chloroplast genome assembly

To perform de novo assembly and compare the seven extant species in the genus *Citrullus*, for *C. rehmii* (SRR8751709), *C. ecirrhosus* (SRR8751725) and *C. naudinianus* (SRR8751817), we used Fastq-dump to download their clean sequence data with 150 bp paired-end reads of total DNA^1^.

Next, the CPGs of the three *Citrullus* species above were assembled using the following method. First, to preclude the influence on chloroplast genome assembly from the consensus reads of nuclear and mitochondria with chloroplasts, 0.8-Gb sequence data were randomly drawn using the Sample function of Seqtk (v0.1) (https://github.com/lh3/seqtk) and then assembled by plasmidspades.py in SPAdes (v3.10.1)^43^. Next, scaffolds with high coverage and length representing the chloroplast genome were extracted and ordered into a draft genome sequence by BLAST against the reference CPG of *C. lanatus* subsp*. vulgaris* (NC_014043.1). Gaps in the chloroplast draft genome sequence of each accession were repaired by GapCloser (v1.12-r6)^44^ and overlap sequences at two flanks of gaps were deleted manually. Finally, the integrity and quality of the chloroplast genome sequence was then inspected and manually corrected by reference-guided mapping using BWA^45^ and SAMtools^46^ untill the CPG was obtained.

### Genome annotation

The three newly assembled CPGs were annotated using CpGAVAS2^47^ and GeSeq^48^, followed by manual adjustments of gene boundaries with errors through inspection using IGV v2.4^49^. The final GenBank and Sequin format of the annotated information was produced in GB2sequin^50^. All records with were then deposited in a Sequin format, and they can be viewed in the NCBI database. GenBank files were created using Sequin v15.50 (http://www.ncbi.nlm.nih.gov/Sequin/index.html) and then drew the chloroplast genome maps in OGDRAW v1.3.1^51^.

### Comparative analysis of genome feature

To explore the intergenic variation among the CPGs of seven *Citrullus* species by genome comparison, the chloroplast reference genomes of *C. lanatus* subsp*. vulgaris*, *C. amarus*, and *C. colocynthis* that we had published in our past researches were also included^14–16^, and the complete chloroplast genome of *C. mucosospermus* were directly obtained from the Organelle Genome Database (OGD; NCBI website). The data for the seven *Citrullus* species were listed in Table 5. The gene rearrangements at the boundary between the single copy (SC) region and inverted repeat (IR) region were inspected and analyzed by IRscope^52^ to understand the variation in binding regions within the CPGs among the seven *Citrullus* species. The chloroplast genome collinear analysis among the seven *Citrullus* species was conducted using Mauve Alignment in Geneious Prime^53^.

**Table 5:** A list of the seven *Citrullus* species used in the study.

Species	Accession	Chloroplast genome ^a^	DNA sequence data accession^b^
*C. rehmii*	Grif16376	–	SRR8751709
*C. ecirrhosus*	Grif16945	–	SRR8751725
*C. naudinianus*	PI 596,694	–	SRR8751817
*C. lanatus* subsp*. vulgaris*	W1-1	NC_032008.1	–
*C. colocynthis*	PI 374,216	NC_035727.1	SRR8751849
*C. amarus*	Grif16135	NC_035974.1	SRR8751536
*C.mucosospermus*	PI 249,010	NC_033899.1	SRR8751721

^a^Plant chloroplast genome directly download from the NCBI ORG database. ^b^Plant whole genome re-sequence data downloaded from the NCBI SRA database according to the accession in Guo et al.^1^

### Sequence divergence and nucleotide diversity analysis

To inspect the CPG sequence divergence of the seven *Citrullus* species, the mVISTA web tool was used to align and visualize the results with default parameters^54^. The CPG of *C. lanatus* subsp*. vulgaris* was used as reference, and then the pairwise distances based on the CPG of seven *Citrullus* species was estimated by MAFFT v7^55^ and MEGA X^56^. Nucleotide diversity in the CPGs of the seven *Citrullus* species were scanned using DNA polymorphism in DNAsp v6.12.03 with an 800-bp window and 200-bp step size^57^. Parsimony-informative base sites (PIC), variable sites and K-2P distance of selected DNA barcodes were analyzed in MEGA v11 software^58^.

### Repeated sequence identification

Dispersed repeats (DR), palindromic repeats (PR), long tandem repeats (TR) (greater than 7 bp), and simple sequence repeats (SSR) were explored in the CPGs of seven *Citrullus* species. Vmatch software was applied to identify the DR and PR with a minimal repeat size of 30 bp, a hamming distance is 3, and a similarity percentage between two repeat copies greater than 90%, the overlapping repeats in the results were merged into one repeat motif by manual correction. TR at least 7 bp in length was detected by Tandem Repeats Finder program with the alignment parameters for match, mismatch, and INDELs set at 2, 7, and 7, respectively^59^. SSRs were determined by MISA with thresholds of 10, 5, 4, 4, 4, and 4 for mono-, di-, tri-, tetra-, penta-, and hexa-nucleotides, respectively^60^.

### Variation calling

To identify the single nucleotide polymorphisms (SNPs) and insertions or deletions (INDELs) of the CPGs between cultivated watermelon (*C. lanatus* subsp*. vulgaris*) and six other *Citrullus* species, we downloaded their total DNA sequence data from the NCBI SRA database, as shown in Table 5. Then, their clean paired reads were aligned to the CPG of *C. lanatus* subsp*. vulgaris* using BWA-mem, and the aligned results were sorted and indexed by SAMtools^45^. Finally, the SNP and INDEL were called by VarScan^61^. Variants with quality scores below 20 and homopolymers were filtered, and the effect of remaining variants in variant call format (VCF) format were annotated and predicted by using SnpEff^62^ based on the transcript definitions of the chloroplast reference genome of *C. lanatus* subsp*. vulgaris*. The high severity effects in the report were chosen to represent the effect of variants.

### Phylogenetic analyses

The phylogenetic tree was built based on the CPG, CDS, SNPs and hypervariable fragments using the maximum likelihood (ML) and Bayesian inference (BI) approaches The CPG of *Cucumis sativus* and *Cucumis melo* served as the out-group. ML was implemented using PhyML^63^ with a GTR + G model; to assess node support, a bootstrap analysis was performed based on 1,000 replicates in UGENE v1.29.0^64^. The BI analysis was conducted in MrBayes v3.2.6^65^, for which the Markov chain Monte Carlo (MCMC) method was run for 1 million generations. Trees were sampled and diagnosed every 1,000 generations, with the first 25% of samples discarded as burn-in. The remaining trees were used to build a 50% majority-rule consensus tree. A steady state was reached if the average standard deviation of the split frequencies was < 0.01. The final phylogenetic tree was edited and visualized in FigTree v1.4.2 software (http://tree.bio.ed.ac.uk/software/figtree/).

### Selective pressure analysis

Protein-encoding genes could be subjected to selective pressure, thus, all consensus protein-encoding genes of the CPGs of the seven *Citrullus* species included in this study were analyzed for selective pressure using Easy-CodeML^66^ and TBtools^67^. In Easy-CodeML with the site model, the nonsynonymous (Ka) and synonymous (Ks) substitution ratios and likelihood ratio test (LRT) values were calculated. The Ka/Ks ratio (ω) and the LRT values were integrated to explore the selective pressure on amino acid sites using naive empirical Bayes and Bayes empirical Bayes methods in the M7 versus M8 model^66^. Meanwhile, in order to determine the selective pressure between *C. lanatus* subsp*. vulgaris* and the other six *Citrullus* species, the Ka/Ks ratios were calculated by the Ka/Ks calculator of TBtools according to their paired protein-encoding genes.

#### SNP validation and SSR marker development

To validate the missense SNP of *petB* between cultivated watermelon and *C. mucosospermus*, the *petB* gene was cloned from W1-1 (*C. lanatus* subsp*. vulgaris*) and PI186490 (*C. mucosospermus*). First, the primer was designed according to the CDS of *petB* in W1-1 (Table 6). Then, total mRNA was extracted from leaves and reverse transcribed for first-strand cDNA synthesis using the M5 Sprint qPCR RT kit according to the manufacturer’s instructions (Mei5 Biotechnology, Co, Ltd). The DNA fragments were amplified and purified according to the operation instructions of the Hieff Canace Plus PCR Master Mix and TIAgel purification kit, and sequenced using the targeted pyrosequencing assay in Sangon Biotech (Shanghai) Co., Ltd. Three selected SSRs with flanking sequences were used to design primers in Primer Premier 5.0^68^ software with default parameters (Table 6). The polymorphism of molecular markers was verified among W1-1 (*C. lanatus* subsp*. vulgaris*), PI186490 (*C. mucosospermus*), and PI269341 (*C. amarus*) using PCR amplification, and PCR assay was conducted according to our previous study^69^. The PCR products were separated with by 6% denaturing polyacrylamide gel electrophoresis (PAGE) and detected by silver staining.

**Table 6:** Designed primer for SNP validation and molecular markers.

Primer name	Forward primer sequence	Forward primer sequence	Tm (℃)	Product size (bp)
petB_p	TTCAGGCGATTGCAGATGAT	TTTCGTGGAGTTGAAGGGAAA	57	742
SSR2	GTACGGCTCGAGGAAATTCA	GGGGTTAGAGACCGCTCAAT	60	238
SSR8	ATGCATTCAGATGGAAAGGC	CCGAACGTGAAACTTTGGTT	60	126
SSR15	GGTTATGCCACGATGTCCTT	GACATGTGGCTTGCTTGGTA	60	280

## Supplementary Information

Supplementary Information 1.

Supplementary Information 2.

## Data Availability

The re-sequencing data of Grif16376 (*C. rehmii*), Grif16945 (*C. ecirrhosus*) and PI596694 (*C. naudinianus*) were in GenBank (https://www.ncbi.nlm.nih.gov/) with accession number SRR8751709, SRR8751725 and SRR8751817, respectively. Fully assembled and annotated plastomes also have been deposited in GenBank (*C. rehmii*: ON597627, *C. ecirrhosus*: MZ577999, *C. naudinianus*: MZ577998).
