# Supplementary material for: Comparative chloroplast genome analysis of seven extant Citrullus species insight into genetic variation, phylogenetic relationships, and selective pressure
Source: Sci Rep. 2023 Apr 25;13:6779. doi: 10.1038/s41598-023-34046-6 (PMC10130142; doi:10.1038/s41598-023-34046-6)
Supplement: Supplementary file 2 — Supplementary Information 2. [file 41598_2023_34046_MOESM2_ESM.pdf]

Comparative chloroplast genome analysis of seven extant *Citrullus* species insight into genetic variation, phylogenetic relationships, and selective pressure

Cong Zhou [1, #], Putao Wang [1, #], Qun Zeng [1], Rongbin Zeng [1], Wei Hu [1], Lei Sun [3], Shi Liu [2], Feishi Luan [2], Qianglong Zhu [1, *]

[1] Jiangxi Key Laboratory of Crop Physiology, Ecology and Genetic Breeding, Jiangxi Agricultural University, Nanchang, 330045, P.R. China

[2] College of Horticulture and Landscape Architecture, Northeast Agricultural University, Harbin, 150030, P.R. China

[3] Department of Agronomy and Horticulture, Liaoning Agricultural Technical College, Yingkou, 115009

[*] Correspondence:

Dr. Qianglong Zhu, Department of Horticulture, College of Agronomy, Jiangxi Agricultural University, NO.1101 Zhimin Street Qingshanhu District, Nanchang, 330045, P.R. China; Tel.: +86 0791-83813185; Fax: +86 0791-83828220; Email: longzhu2011@126.com

# These authors contributed equally to this study

Supplementary Information:

Figure S1. The variation of IR/SC boundary of seven *Citrullus* species. The genes on the positive chain were painted up the tracks representing each accession, while the genes on the negative chain were painted down the tracks representing each accession. The number on the arrow indicates the distance between the gene start or end position and the joint point of boundary. The abbreviations in the figure indicate junction sites in the chloroplast genomes: JLA (IRa/LSC), JLB (IRb/LSC), JSA (SSC/IRa) and JSB (IRb/SSC). Genes are drawn by different color boxes. The figure is not to scale by sequence length.

Figure S2. The chloroplast genome collinear analysis among the seven Citrullus species by Mauve Alignment.

Figure S3. Phylogenetic relationship analysis based on the five hypervariable fragments using maximum likelihood (ML) with 1000 bootstrap value.

Figure S4. The full-length gels for selecting SSR makers with polymorphism among different watermelon germplasm resource. From left to right, the frist three makers were SSR2, SSR8 and SSR15, respectively, the samples were LSW177, W1-1, PI186490, PI269341, W1-60, respectively.

Figure S5. Phylogenetic relationship analysis based on the CDS, SNP and rps16-trnQ of seven *Citrullus* species using the maximum likelihood (ML) and Bayesian inference (BI). The corresponding ML-BS/BI-PP values are given at each node.

Figure S6. Phylogenetic relationship analysis based on the complete chloroplast genome of C. rehmii (PI632755) and other seven Citrullus species using the maximum likelihood (ML) and Bayesian inference (BI). The corresponding ML-BS/BI-PP values are given at each node.

Table S1. Estimates of evolutionary divergence between sequences.

Table S2. The Pi value of 79 consensus protein-coding genes.

Table S3. The number of TR, PR, DR, and SSRs in all seven *Citrullus* species chloroplast genomes.

Table S4. The distribution of total repeats in the chloroplast genomes of seven Citrullus species.

Table S5. The average number and proportions of mono-, di-, tri-, and tetra-nucleotide SSRs.

Table S6. The consensus polymorphic SSR makers among seven *Citrullus* species.

Table S7. The effect of SNP in the chloroplast genomes of seven *Citrullus* species.

Table S8. The effect of INDEL in the chloroplast genomes of seven *Citrullus* species.

Table S9. The Ka/Ks of pair species in *Citrullus*.

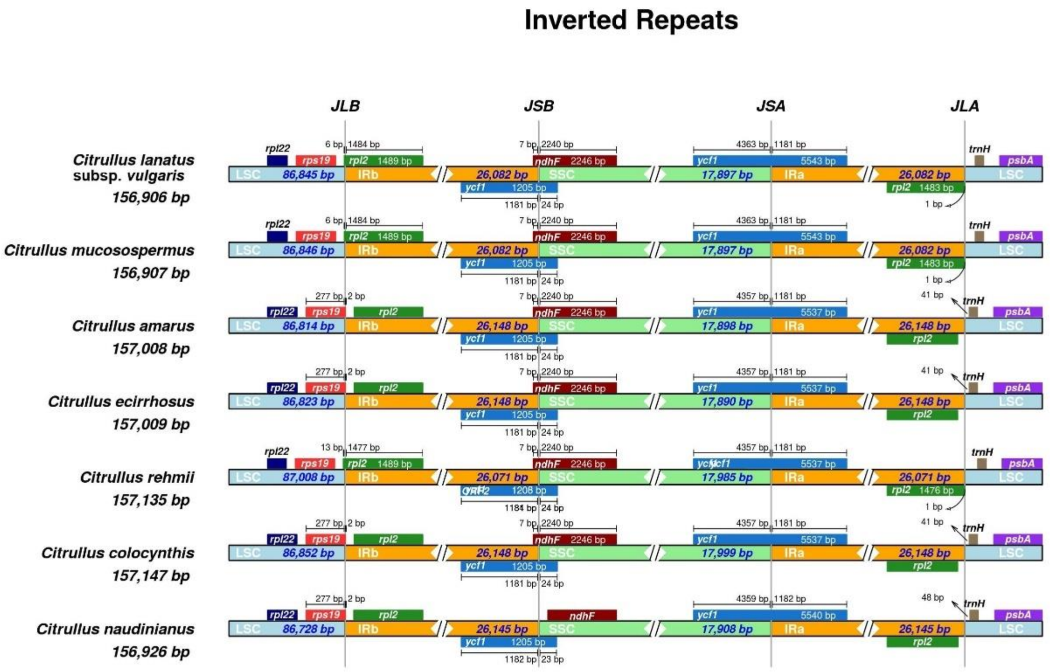

Figure S1. The variation of IR/SC boundary of seven Citrullus species. The genes on the positive chain were painted up the tracks representing each accession, while the genes on the negative chain were painted down the tracks representing each accession. The number on the arrow indicates the distance between the gene start or end position and the joint point of boundary. The abbreviations in the figure indicate junction sites in the chloroplast genomes: JLA (IRa/LSC), JLB (IRb/LSC), JSA (SSC/IRa) and JSB (IRb/SSC). Genes are drawn by different color boxes. The figure is not to scale by sequence length.

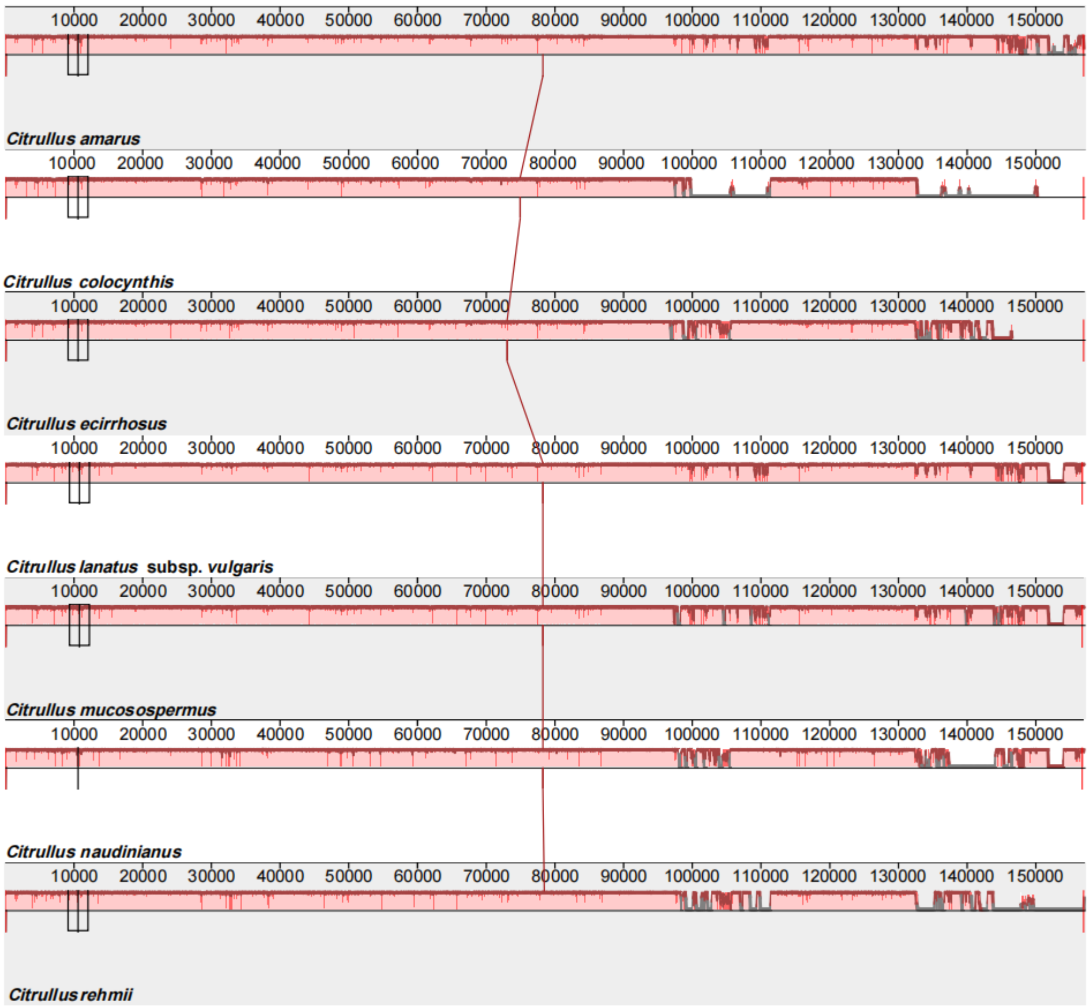

Figure S2. The chloroplast genome collinear analysis among the seven Citrullus species by Mauve Alignment.

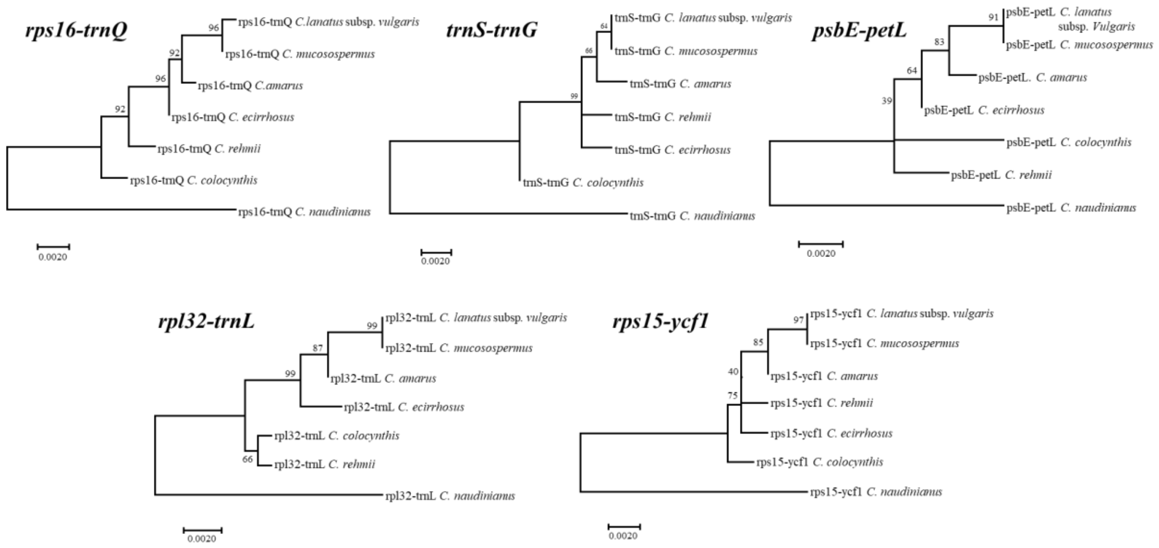

Figure S3. Phylogenetic relationship analysis based on the five hypervariable fragments using maximum likelihood (ML) with 1000 bootstrap value.

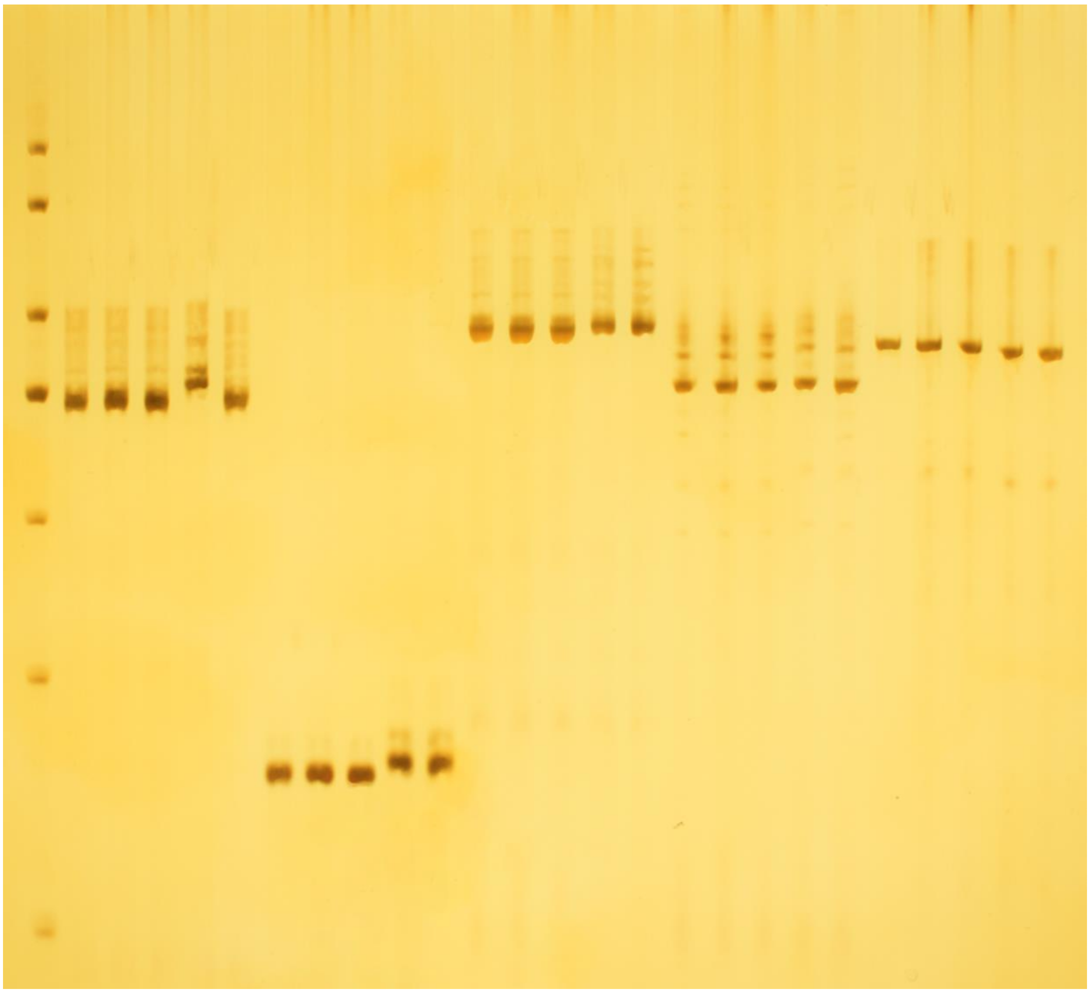

Figure S4. The full-length gels for selecting SSR makers with polymorphism among different watermelon germplasm resource. From left to right, the first three makers were SSR2, SSR8 and SSR15, respectively, the samples were LSW177, W1-1, PI186490, PI269341, W1-60, respectively.

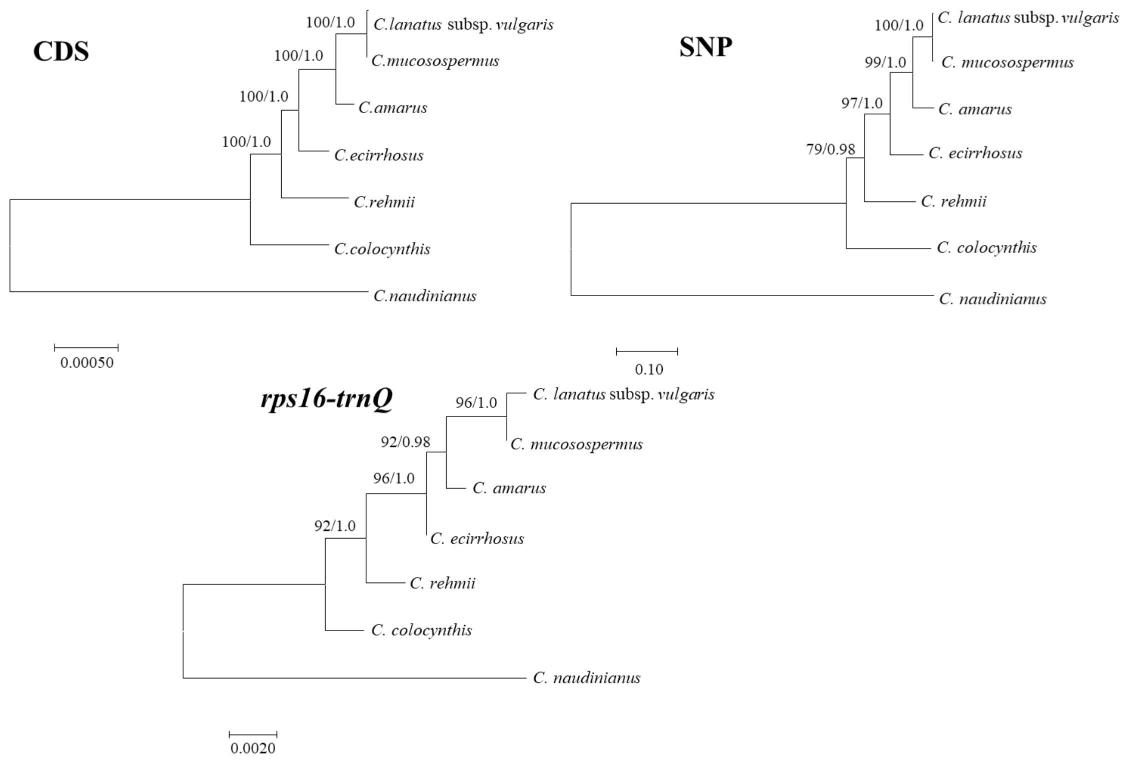

Figure S5. Phylogenetic relationship analysis based on the CDS, SNP and rps16-trnQ of seven *Citrullus* species using the maximum likelihood (ML) and Bayesian inference (BI). The corresponding ML-BS/BI-PP values are given at each node.

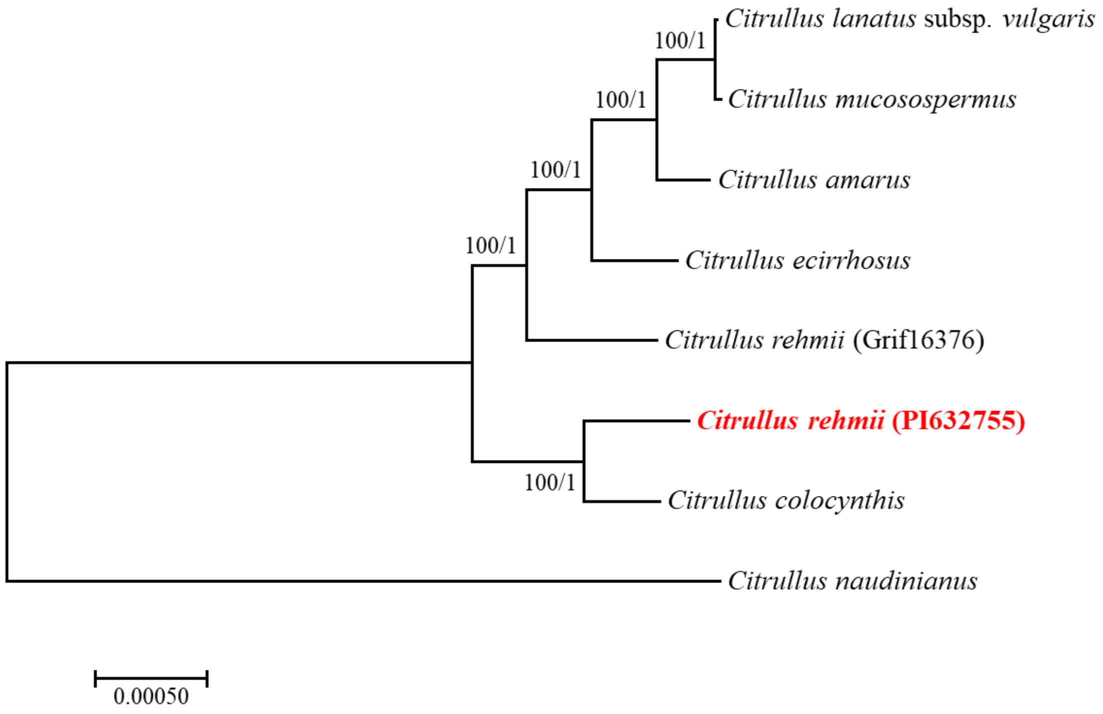

Figure S6. Phylogenetic relationship analysis based on the complete chloroplast genome of C. rehmii (PI632755) and other seven Citrullus species using the maximum likelihood (ML) and Bayesian inference (BI). The corresponding ML-BS/BI-PP values are given at each node.

Table S1. Estimates of Evolutionary Divergence between Sequences

	Citrullus lanatus subsp. *vulgaris*	*Citrullus mucosospermus*	*Citrullus amarus*	*Citrullus ecirrhosus*	*Citrullus rehmii*	*Citrullus colocynthis*	*Citrullus naudinianus*
Citrullus lanatus		0.00001	0.00003	0.00008	0.00006	0.00006	0.00014
Citrullus mucosospermus	0.00004		0.00004	0.00008	0.00007	0.00006	0.00013
Citrullus amarus	0.00051	0.00052		0.00008	0.00006	0.00006	0.00014
Citrullus ecirrhosus	0.00096	0.00097	0.00091		0.00007	0.00005	0.00014
Citrullus rehmii	0.00146	0.00147	0.00141	0.00126		0.00005	0.00014
Citrullus colocynthis	0.00190	0.00191	0.00186	0.00178	0.00168		0.00017
Citrullus naudinianus	0.00640	0.00641	0.00639	0.00619	0.00611	0.00613	

Table S2. The Pi value of 79 consensus protein-coding genes

Gene name	Pi value	Gene name	Pi value
rpl32	0.00754	psbD	0.00135
ycf1	0.00664	psbH	0.00129
psbK	0.0062	rpoC1	0.00126
clpP1	0.00617	cemA	0.00124
petL	0.006	rbcL	0.0012
ndhF	0.00503	atpH	0.00116
accD	0.00371	pafII	0.00103
rps4	0.00363	ndhE	0.00094
atpB	0.00358	rps14	0.00094
rps8	0.00354	psaB	0.00091
rpoC2	0.00349	ndhK	0.00084
rps3	0.00306	psbA	0.00081
atpF	0.00293	rpl14	0.00078
rpoA	0.00286	atpI	0.00077
rpl33	0.00285	psbB	0.00075
rps2	0.00282	atpE	0.0007
rps15	0.0028	pafI	0.00056
rps11	0.00275	ycf2	0.0004
ndhD	0.00273	ndhB	0.00037
ndhG	0.0027	rpl2	0.00035
psbI	0.00258	infA	0
rpl16	0.00258	rps19	0
rpl36	0.00251	pbf1	0
ndhC	0.00237	petG	0
petD	0.00237	petN	0
ndhA	0.00236	psaC	0
ndhH	0.00226	psaI	0
rpl22	0.00226	psbE	0
ndhI	0.00225	psbF	0
petB	0.00221	psbJ	0
psaJ	0.00212	psbL	0
matK	0.00208	psbM	0
psbC	0.00207	psbT	0
petA	0.00198	psbZ	0
atpA	0.00188	rpl23	0
ndhJ	0.0018	rps12	0
ccsA	0.00177	rps16	0
psaA	0.00169	rps18	0
rpl20	0.00162	rps7	0
rpoB	0.00146	-	-

Table S3. The number of TR, PR, DR, and SSRs in all seven *Citrullus* species chloroplast genomes.

Repeat types	*Citrullus lanatus* subsp. *vulgaris*	*Citrullus mucosospermus*	*Citrullus amarus*	*Citrullus ecirrhosus*	*Citrullus rehmii*	*Citrullus colocynthis*	*Citrullus naudinianus*
TR	21	21	21	19	21	20	27
PR	19	19	18	21	19	22	19
DR	11	11	11	13	13	13	13
SSR	56	55	57	57	53	54	56
total number	107	106	107	110	106	109	115

Table S4. The distribution of total repeats in the chloroplast genomes of seven Citrullus species

Genomic region	*Citrullus lanatus* subsp. *vulgaris*	*Citrullus mucosospermus*	*Citrullus amarus*	*Citrullus ecirrhosus*	*Citrullus rehmii*	*Citrullus colocynthis*	*Citrullus naudinianus*
Intergenic	58	59	65	63	59	60	62
Intron	16	15	12	11	11	11	11
Exon	33	32	30	36	36	38	42
Total number	107	106	107	110	106	109	115

Table S5. The average number and proportions of mono-, di-, tri-, and tetra-nucleotide SSRs

Unit size	*Citrullus lanatus* subsp. *vulgaris*	*Citrullus mucososperm us*	*Citrullus amarus*	*Citrullus ecirrhosus*	*Citrullus rehmii*	*Citrullus colocynthi s*	*Citrullus naudinianus*	Average (rate)
mono	40	39	40	40	37	37	45	40 (71.6%)
di	12	12	13	13	12	13	7	12 (21.1%)
tri	2	2	2	2	2	2	2	2 (3.6%)
tetra	2	2	2	2	2	2	2	2 (3.6%)
Total	56	55	57	57	53	54	56	56

Table S6. The consensus polymorphic SSR makers among seven Citrullus species

SSR label	Core motif	Allelic constitution						
		C. lanatus subsp. *vulgaris*	*C. mucosospermus*	*C. amarus*	*C. ecirrhosus*	*C. rehmii*	*C. colocynthis*	*C. naudinianus*
SSR1	(A)n	15	15	16	14	11	12	9
SSR2	(A)n	12	12	12	14	6	6	6
SSR3	(A)n	10	10	10	10	5	10	9
SSR4	(A)n	12	12	12	10	11	9	9
SSR5	(A)n	13	13	13	13/14	13/14	12	16
**SSR6**	(ATAA)n	3	3	3	3	3	3	3
SSR7	(T)n	10	10	9	9	9	9	5
SSR8	(T)n	10	10	14	10	11	10	10
SSR9	(A)n	10	9	9	9	9	9	8
SSR10	(T)n	12	12	11	11	12	10	10
SSR11	(T)n	16	16	10	10	10	9	10
**SSR12**	(T)n	13	13	13	13	13	13	13
**SSR13**	(AT)n	5	5	5	5	5	5	5
**SSR14**	(AAAT)n	3	3	3	3	3	3	3
SSR15	(T)n	11	11	12	14	10	9	11
SSR16	(T)n	10	10	10	10	10	10	9
**SSR17**	(TTTA)n	3	3	3	3	3	3	3
SSR18	(T)n	11	12	9	11	11	11	9
SSR19	(TA)n	6	6	6	6	6	6	4
**SSR20**	(TAAT)n	3	3	3	3	3	3	3
SSR21	(ATTTG)n	3	3	2	2	2	2	2

SSR22	(A)n	13	13	12	13	13	13	12
SSR23	(A)n	10	10	9	10	10	10	9
SSR24	(A)n	12	11	13	11	13	11	11
SSR25	(T)n	11	11	10	11	11	10	13
SSR26	(T)n	10	10	11	12	11	11	10
SSR27	(T)n	12	12	12	12	12	12	10
SSR28	(T)n	14	14	11	11	11	10	9
SSR29	(T)n	10	10	10	10	9	9	9
SSR30	(A)n	15	15	15	14	15	15	12
SSR31	(T)n	13	13	12	12	11	10	13
SSR32	(T)n	13	13	12	11	12	12	10
SSR33	(A)n	14	14	12	15	14	12	9
SSR34	(T)n	10	10	11	9	9	12	8
**SSR35**	(ATTT)n	4	4	4	4	4	4	4
**SSR36**	(AAAT)n	3	3	3	3	3	3	3
**SSR37**	(A)n	10	10	10	10	10	10	10
**SSR38**	(AAGT)n	3	3	3	3	3	3	3
**SSR39**	(TTA)n	4	4	4	4	4	4	4
SSR40	(T)n	10	10	10	9	9	10	9
SSR41	(TA)n	5	5	5	5	5	6	4
SSR42	(T)n	11	11	13	13	12	11	10
**SSR43**	(CAAA)n	3	3	3	3	3	3	3
SSR44	(TC)n	5	5	5	5	5	5	4
SSR45	(T)n	10	10	10	10	10	6	10
SSR46	(A)n	11	11	11	11	9	11	11
**SSR47**	(AAAT)n	4	4	4	4	4	4	4
